# C-Type Lectin Receptor Mediated Modulation of T2 Immune Responses to Allergens

**DOI:** 10.1007/s11882-023-01067-0

**Published:** 2023-02-01

**Authors:** Alba Angelina, Leticia Martín-Cruz, Andrés de la Rocha-Muñoz, Begoña Lavín-Plaza, Oscar Palomares

**Affiliations:** 1 Department of Biochemistry and Molecular Biology, School of Chemistry, Complutense University of Madrid, Avenida Complutense S/N, Madrid, 28040 Spain; 2 Autonomous University of Madrid, Madrid, Spain

**Keywords:** C-type lectin receptors (CLRs), Type-2 immune responses, Allergy, Allergens, Allergen-specific immunotherapy (AIT), Mannan

## Abstract

**Purpose of Review:**

Allergic diseases represent a major health problem of increasing prevalence worldwide. In allergy, dendritic cells (DCs) contribute to both the pathophysiology and the induction of healthy immune responses to the allergens. Different studies have reported that some common allergens contain glycans in their structure. C-type lectin receptors (CLRs) expressed by DCs recognize carbohydrate structures and are crucial in allergen uptake, presentation, and polarization of T cell responses. This review summarizes the recent literature regarding the role of CLRs in the regulation of type 2 immune responses to allergens.

**Recent Findings:**

In this review, we highlight the capacity of CLRs to recognize carbohydrates in common allergens triggering different signaling pathways involved in the polarization of CD4^+^ T cells towards specific Th2 responses. Under certain conditions, specific CLRs could also promote tolerogenic responses to allergens, which might well be exploited to develop novel therapeutic approaches of allergen-specific immunotherapy (AIT), the single treatment with potential disease-modifying capacity for allergic disease. At this regard, polymerized allergens conjugated to non-oxidized mannan (allergoid-mannan conjugated) are next-generation vaccines targeting DCs via CLRs that promote regulatory T cells, thus favoring allergen tolerance both in preclinical models and clinical trials.

**Summary:**

A better understanding of the role of CLRs in the development of allergy and in the induction of allergen tolerance might well pave the way for the design of novel strategies for allergic diseases.

## Introduction

Our immune system orchestrates type 2 immune responses to protect us against helminths, venoms, and toxins. However, under certain conditions, aberrant type 2 immune responses arise leading to different type 2 inflammatory disorders such as allergic diseases. Allergy represents a global health problem of increasing prevalence that affects around 30% of the population worldwide [1, 2]. Type 2–mediated allergic diseases include allergic rhinitis, allergic asthma, atopic dermatitis, food allergy, and anaphylaxis [2]. Dendritic cells (DCs) are professional antigen-presenting cells essential for the generation of proper immune responses [3]. In the context of allergy, DCs recognize and internalize encountered allergens within the skin and respiratory and gastrointestinal tracts, which are processed and then presented to naïve CD4^+^ T cells in the lymph nodes. Depending on the antigens and the signals that DCs receive in the peripheral tissues and in the lymph nodes, antigen presentation might trigger the induction of potent allergen-specific Th2 responses or regulatory T (Treg) cells, thus promoting allergic inflammation or allergen tolerance, respectively [4–6]. DCs are equipped with a large number of pattern recognition receptors (PRRs) including Toll-like receptors (TLRs) and C-type lectin receptors (CLRs) among others [7, 8]. CLRs recognize a wide range of carbohydrate structures contained in allergens, which might influence allergen uptake, subsequent processing, and presentation as well as specific T cell polarization [4, 9]. The final functional outcomes depend not only on the specific targeted CLRs but also on the physical nature, affinity, and avidity of the carbohydrate ligands [7]. A quick expansion in the understanding of the role of CLRs in the context of allergy has taken place over the last years [10••]. Some allergens from common allergenic sources such as house dust mite (HDM), peanut, or animal dander contain glycan structures recognized by specific CLRs, which in turn promote allergic responses [11]. On the other hand, compelling experimental evidence also demonstrated that triggering of specific CLRs might induce immune regulation and allergen tolerance [12]. At this regard, allergoids conjugated to mannan are next-generation vaccines for allergen-specific immunotherapy (AIT) targeting DCs that are able to induce allergen tolerance [12, 13]. Allergoid-mannan conjugates have demonstrated efficacy and safety in phase II trials and are currently under evaluation in phase III clinical trials [14••]. Herein, we review and provide an update on the role of CLRs in the regulation of type 2 immune responses to allergens and discuss the most relevant findings related to the potential therapeutic applications of CLR ligands for treatment of allergic diseases.

## Structural and Functional Feature of C-Type Lectin Receptors

**Fig. 1:**
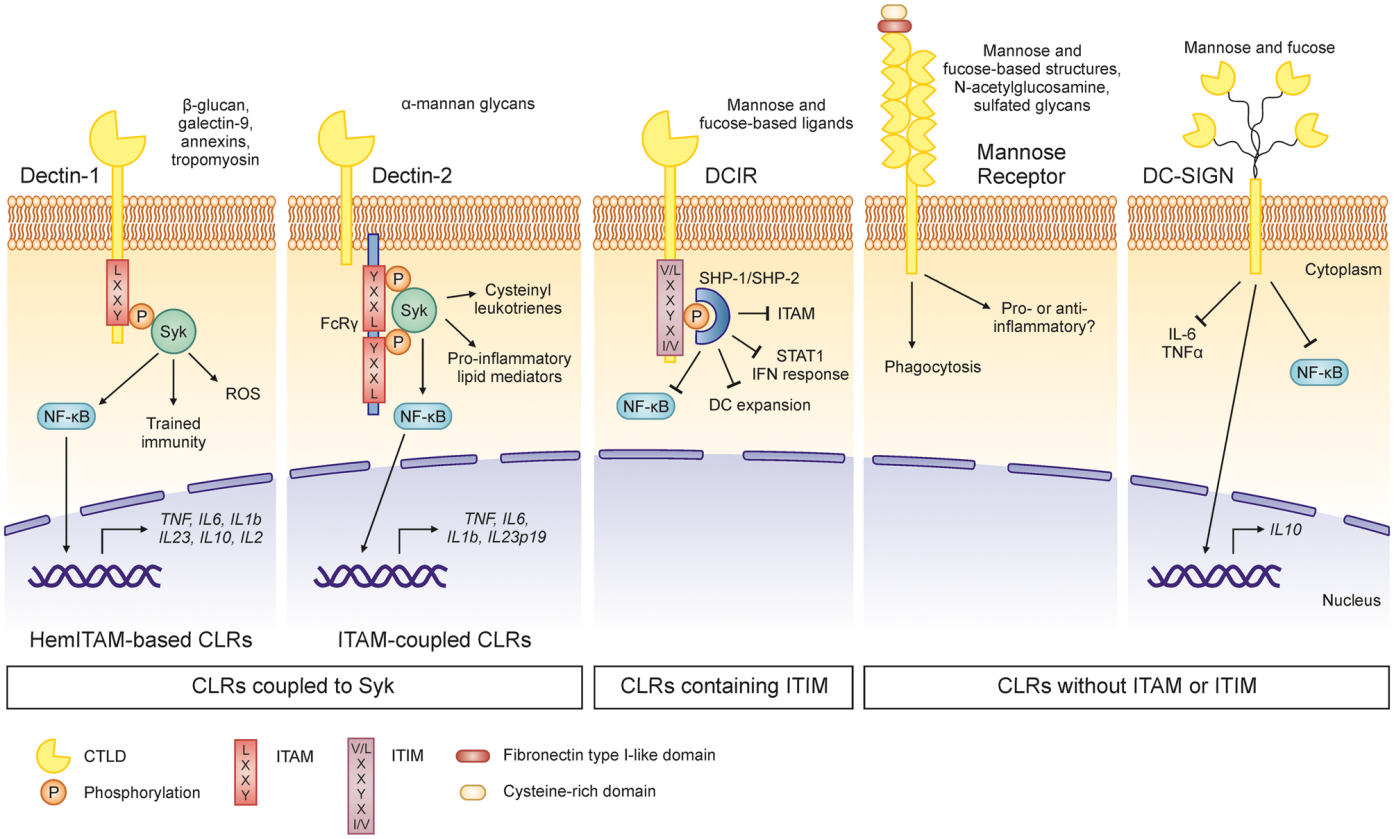
Main CLR-induced signaling pathways. The recognition of fungal β-glucan, galectin-9, annexins, or tropomyosin by dectin-1 induces phosphorylation (P) of the immunoreceptor tyrosine–based activation motif (ITAM) which facilitates the recruitment of spleen tyrosine kinase (Syk). Subsequently, activation of canonical and non-canonical nuclear factor-κB (NF-κB) results in the induction of *TNF*, *IL6*, *IL1b*, *IL23*, *IL10*, and *IL2* gene expression. Dectin-1 triggering also promotes reactive oxygen species (ROS) and the specific recognition of β-glucans induces trained immunity. Dectin-2 recognizes α-mannan glycans which induce signaling pathways through ITAM containing the adaptor molecule (e.g., Fc receptor γ‑chain (FcRγ)). The phosphorylation of ITAM recruits Syk and induces NF-κB signaling pathway modulating *TNF*, *IL6*, *IL1b*, and *IL23p19* gene expression. Dectin-2-mediated activation of Syk is involved in the production of cysteinyl leukotrienes and pro-inflammatory lipid mediators. Activation of dendritic cell (DC) immunoreceptor (DCIR) by mannose and fucose-based ligands leads to the phosphorylation of the immunoreceptor tyrosine–based inhibitory motifs (ITIM). The recruitment of the phosphatases SH2‑domain‑containing protein tyrosine phosphatase 1 (SHP1) or SHP2 inhibits NF-κB and STAT1-type I IFN signaling pathways, and limits DC expansion and downregulation of ITAM. Mannose receptor and DC-specific ICAM3‑grabbing nonintegrin (DC-SIGN) lack ITAM or ITIM domains. Following the recognition of mannose and fucose-based structures, N-acetylglucosamine, or sulfated glycans, mannose receptor mediates phagocytosis. Its implication in pro- or anti-inflammatory responses is still not clear. DC-SIGN binds mannose and fucose inducing the inhibition of the NF-κB signaling pathway that mediates the downregulation of IL-6 and TNFα production, whereas favoring *IL10* gene expression

The CLRs are a superfamily of receptors characterized by the presence of at least one C-type lectin-like domain (CTLD), which is defined by its ability to bind carbohydrates in a calcium-dependent manner [15]. The CLR superfamily is formed by more than 1000 members, organized in 17 groups, and recognizes self- and non-self-ligands involved in different functions such as cell adhesion, phagocytosis, homeostasis, and innate and adaptive immunity [7, 16]. Ligand recognition occurs generally through their extracellular CTLD which is tightly regulated by specific amino acid motifs, calcium ions, and the carbohydrate structure [17]. Based on the cytoplasmic signaling motifs and the signaling pathways triggered upon ligand binding, myeloid CLRs can be classified in three categories (Fig. 1): CLRs coupled to the spleen tyrosine kinase (Syk), CLRs containing immunoreceptor tyrosine–based inhibitory motifs (ITIM), and CLRs without immunoreceptor tyrosine–based activating motif (ITAM) or ITIM domains [16]. In this review, we will focus on membrane-associated CLRs that are mainly expressed in myeloid cells (Table 1) and specifically on dectin-1, dectin-2, DC immunoreceptor (DCIR), mannose receptor (MR), and DC-specific intercellular adhesion molecule 3-grabbing nonintegrin (DC-SIGN) as CLRs involved in the regulation of type 2 immune responses to allergens (Fig. 1) [10••].

**Table 1:** Main C-type lectin receptors (CLRs), localization, and ligands

**Receptor**	**Gene name**	**Localization**	**Ligand**
**CLRs coupled to the spleen tyrosine kinase (Syk): HemITAM-based CLRs**			
Dectin-1 (β-GR, CLECSF12)	*CLECL7A (Hu)* *Clec7a (Ms)*	DCs, monocytes, macrophages, neutrophils, γδ T cells, B cells, eosinophils, mast cells, epithelium	β-1,3 Glucans, galectin-9, annexins, tropomyosin
DNGR-1	*CLECL9A (Hu)* *Clec9a (Ms)*	BDCA-3^+^ DCs (Hu), CD8α^+^ DCs, tissue resident CD103 CD11b^−^ DCs	ND
SIGNR3	*Cd209d (Ms)*	Macrophages	High mannose, fucose
**CLRs coupled to the spleen tyrosine kinase (Syk): ITAM-coupled CLRs**			
Dectin-2	*CLEC6A (Hu)* *Clec4n (ms)*	Macrophages, monocytes, several DC subtypes	High mannose, *a-*mannans
BDCA-2 (DLEC, CD303, CLECSF7)	*CLEC4C (Hu)*	Plasmacytoid DCs	gp120
DCAR	*Clec4b1 (Ms)*	DCs, monocytes, B cells, macrophages	ND
Mincle	*CLEC4E (Hu)* *Clec4e (Ms)*	Macrophages, neutrophils	*a-*Mannose, glycolipids, SAP-130
MDL-1 (CLECSF5)	*CLEC5A (Hu)* *Clec5a (Ms)*	Monocytes, macrophages, osteoclasts	ND
**CLRs containing ITIM**			
DCIR (CLECS-F6, LLIR) Dcir1 (DCIR, Clecf6) Dcir2 (33D1)	*CLEC4A (Hu)* *Clec4a2 (Ms)* *Clec4a4 (Ms)*	Monocytes, B cells, macrophages, DCs, granulocytes	Mannose, fucose
MICL (DCAL-2, KLRL1, CLL1)	*CLECL12A (Hu)* *Clec12a (Ms)*	Granulocytes, DCs, monocytes, macrophages	ND
Macrophage antigen H (CLECL12B)	*CLECL12B (Hu)* *Clecl12b (Ms)*	Macrophages	Surface ligands upregulated following DNA damage
**CLRs without ITAM or ITIM**			
Mannose receptor (MR, MMR, CD206)	*MRC1 (Hu)* *Mrc1 (Ms)*	Macrophages, monocyte-derived DCs, smooth muscle cells, epithelial cells, mesangial cells	High mannose, fucose, sLex, N-acetyl glucosamine, dulfated glycans
DC-SIGN (CLEC4L)	*CD209 (Hu)*	DCs, macrophages	High mannose, fucose
DEC-205 (CD205)	*LY75 (Hu)* *Ly75 (Ms)*	CD8a^+^ DCs, B cells, macrophages, T cells, granulocytes	PLA (*Y. pestis*), K12 (*E. coli*)
SIGNR1	*Cd209b (Ms)*	Macrophages, lamina propria DCs	Dextran, mannan, fucose

### Dectin-1

Dectin-1 is a type II transmembrane protein classified as HemITAM-based CLRs as it contains half of the classical ITAM (YXXL tandem repeats), in their intercellular tail allowing direct binding to Syk (Fig. 1) [16]. Dectin-1 was described to bind β-glucans, commonly found in the cell wall of plants, fungi, and some bacteria. Other ligands such as galectin-9, annexins, or tropomyosin, among others, have been also identified, suggesting dectin-1 as one of the most versatile receptors in myeloid cells [18]. The protein surface residues Trp221 and His223 are key in the ligand binding in a calcium independent manner [19]. Dectin-1 is expressed in DCs, monocytes, macrophages, neutrophils, B cells, and the Gr-1^+^ subset of splenic T cells in mice (Table 1) [10••]. Similar expression pattern has been described for human, including expression in eosinophils and mast cells [19]. Dectin-1 displays a single CTLD connected by a stalk to a single transmembrane region, and a cytoplasmic tail with the HemITAM (Fig. 1). Phosphorylation of the tyrosine residue generates docking sites for the SH2 Syk domains, triggering a conformational change and activation through autophosphorylation [16]. Syk can phosphorylate several molecules inducing a plethora of molecular pathways involved in pro-inflammatory responses, such as cytokine production, reactive oxygen species (ROS) generation, phagocytosis, and trained immunity [16, 18, 20].

### Dectin-2

Dectin-2 is a type II transmembrane protein classified as ITAM-coupled CLRs [16]. Dectin-2 has high affinity for α-mannan glycan structures and binding occurs in a calcium-dependent fashion [10••]. Moreover, dectin-2 has been shown to have a putative endogenous ligand on CD4^+^ T cells and to recognize the HDM allergens [17]. Dectin-2 is expressed in macrophages and some DC subsets in both mouse and human (Table 1) [10••]. Dectin-2 is formed by a single extracellular CTLD connected by a neck region to a short cytoplasmic tail that lacks a clear intracellular signaling motif [17]. Association of dectin-2 with adaptor proteins, such as the Fc receptor γ (FcRγ) chain or DNAX-activation protein 12 (DAP12) that contain ITAM, is required for the engagement to Syk (Fig. 1) [16]. Similar to dectin-1, several signaling pathways are induced via dectin-2, highlighting the expression of cysteinyl leukotrienes, pro-inflammatory lipid mediators, and the Th17 polarizing cytokines IL-1β and IL-23 in DCs [16, 21].

#### DCIR

DCIR is a type II transmembrane protein classified as an ITIM-containing CLR and shows specificity for mannose- and fucose-based glycans and its binding is modulated by the glycosylation of the CTLD [22]. DCIR is expressed in human monocytes, macrophages, granulocytes, B cells, and DCs (Table 1) [16]. Four homologs have been described in mice, highlighting Dcir1 which is expressed in B cells, monocytes/macrophages, and DCs [16]. DCIR structure has a single CTLD followed by neck and transmembrane domains that connect with the cytoplasmic tail that has an ITIM which upon phosphorylation of the tyrosine residue transduces inhibitory signals by activating phosphatases SHP-1 and SHP-2 [23]. DCIR inhibits nuclear factor-κB (NF-κB) signaling, limits DC expansion, and activates the STAT1-type I IFN signaling, controlling differentiation of T lymphocytes towards Th1 [16, 24].

### Mannose Receptor and DC-SIGN

Mannose receptor (MR) was one of the first CLRs discovered. MR is a type I integral transmembrane glycoprotein that recognizes glycans such as mannose- and fucose-based structures, N-acetylglucosamine, and sulfated glycan structures in a calcium-dependent manner [10••, 16, 17]. MR expression was originally restricted to resident macrophages, but now it is known to be also expressed in monocyte-derived DCs, lymphatic and hepatic epithelium, kidney mesangial cells, tracheal smooth muscle cells, and retinal pigment epithelium (Table 1) [16, 19]. MR has three regions, a cysteine-rich domain, a fibronectin type II–like domain that binds collagen, and 8 CTLDs, which forms the extracellular part, followed by a transmembrane region and a cytoplasmic tail that is involved in receptor internalization and recycling (Fig. 1) [17]. Importantly, like DC-SIGN, MR lacks ITAM or ITIM [17]. MR has been implicated in pathogen phagocytosis via the activation of the complex Rac-1, Cdc42, and PAK-1 [17]. Activation of MR promotes anti-inflammatory properties in immature monocyte-derived DCs by inducing IL-10 and IL-1Ra production [16]. However, MR-deficient macrophages produce less TNFα and more IL-10 [16]. These differences might strongly depend on the nature, affinity, and avidity of ligand binding, but further research is still needed to better clarify the induced signaling pathways.

DC-SIGN (CD209) is a type II transmembrane protein and binds, in a calcium-dependent manner, a broad spectrum of mannose and fucose ligands found in viruses, parasites, fungi, bacteria, and self-ligands such as intercellular adhesion molecule (ICAM)-2, ICAM-3, and Mac-1 [17]. It is expressed in immature and mature monocyte-derived DCs, dermal DCs, and macrophages (Table 1) [10••]. DC-SIGN is arranged as a tetramer with autonomous CTLD that interacts through an α-helix neck domain, followed by a transmembrane region and a cytoplasmic domain [15]. The signaling cascade affected by DC-SIGN depends on the type of ligand that binds. DC-SIGN does not induce an immediate downstream signaling; however, it has been shown to modulate the response initiated by the TLRs [17]. In addition, an intracellular signalosome can be formed inducing, on one side, anti-inflammatory cytokines such as IL-10 and suppressing and, on the other side, pro-inflammatory responses via acetylation of the NF-κB subunit p65 and production of IL-6 and TNFα (Fig. 1) [17].

## The Role of CLR in the Context of Allergic Diseases

Allergic diseases are mainly type 2 immune-mediated disorders characterized by the formation of specific IgE antibodies against innocuous substances called allergens. Allergic sensitization occurs after the first contact with the allergen, which is captured by DCs in the airways, gut, or skin. DCs process and transport the allergen to the drain lymph nodes where it is presented to naïve CD4^+^ T cells, leading to the generation of allergen-specific CD4^+^ Th2 cells. After IgE class-switching, B cells produce high amounts of allergen-specific IgE that bind to the high-affinity FcɛRI on mast cells and basophils, thus leading to patient’s sensitization [2, 25]. New allergen encounters trigger cross-linking of the IgE-FcɛRI complexes on mast cells and basophils, inducing the release of the anaphylactogenic mediators that are responsible for the immediate clinical symptoms. Memory allergen-specific Th2 cells activated via IgE-facilitated allergen presentation by DCs and B cells and type 2 innate lymphoid cells (ILC2) activated by epithelial cell–derived alarmins (TSLP, IL-33, and IL-25) produce large amounts of IL-4, IL-5, IL-9, and IL-13 contributing to maintain allergen-specific IgE levels, eosinophilia, mucus production, recruitment of inflammatory cell, and tissue inflammation, which are involved in the chronicity and the most severe clinical manifestations of allergy [2, 25].

The initiation of type 2 immune response prompted by allergens entails its uptake and processing by antigen-presenting cells, mainly DCs, a process that might be enhanced by specific CLRs. Common allergens such as Der p 1, Der p 2, Fel d 1, Ara h 1, Bla g 2, or Can f 1 are glycoproteins, many of which contain structurally related oligosaccharides. Compelling experimental evidence show that the common oligosaccharide structure on allergens is a pentasaccharide core, Man3-GlcNAc2, which often contains additional monosaccharides [11, 26]. CLRs, including dectin-1, dectin-2, DCIR, MR, and DC-SIGN, express on DCs and other myeloid immune cells can recognize carbohydrate structures on allergens, enhancing their uptake and also triggering different downstream signaling responses that might well promote type 2–mediated responses or tolerance depending on the specific CLRs engaged, the subsequent signaling pathways initiated, and the physical nature of the carbohydrate ligands as well as on the affinity and avidity of the carbohydrate-CLR interaction [7, 27]. In the next paragraphs, we will summarize the most recent findings related to the role of specific CLRs that have been involved in the recognition of different allergens and how this might regulate the initiation and maintenance of type 2 immune responses in the context of allergic diseases.

**Fig. 2:**
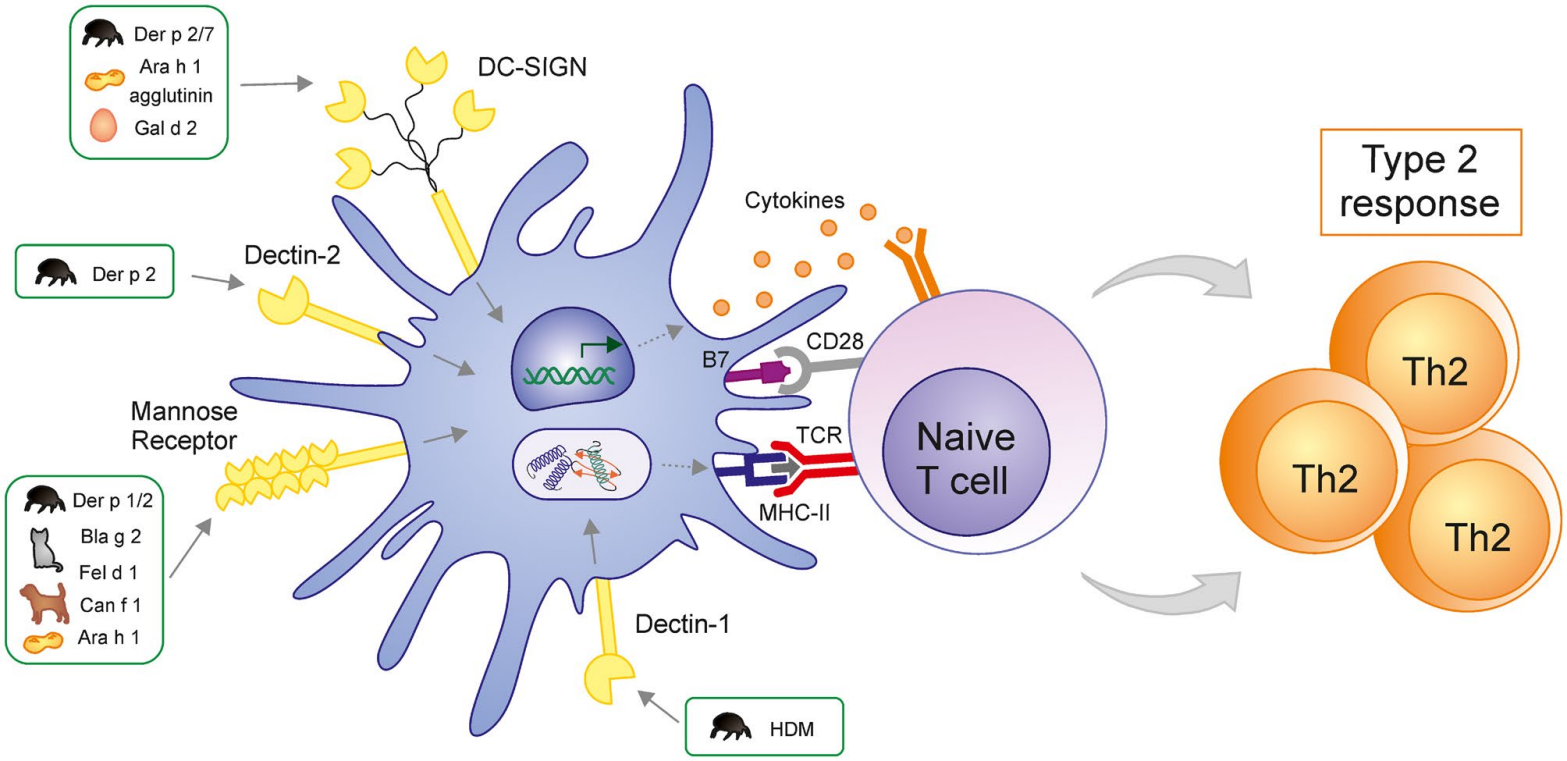
CLR signaling in DCs drives Th2 responses. Allergens such as Der p 1 and Der p 2 from HDM (house dust mite), Can f 1 from dogs, Ara h 1 from peanuts, Bla g 2 from cockroaches, and Fel d 1 from cats are recognized and captured by C-type lectin receptors (CLRs) on dendritic cells (DCs) through their carbohydrates motifs and promote the polarization towards Th2 cells. DC-SIGN, DC-specific intercellular adhesion molecule 3-grabbing nonintegrin; TCR, T cell receptor; MHC-II, major histocompatibility complex class II

There are conflicting data about the role of dectin-1 in allergy. Murine models of HDM- and ovalbumin-induced airway inflammation have demonstrated that dectin-1-deficient (Clec7a^−/−^) mice displayed less eosinophilic and neutrophilic inflammation as well as Th2 and Th17 cells compared with wild-type mice [28, 29]. Dectin-1 expressed by CD11b^+^ DCs senses some glycans in the HDM extract and promotes allergic airway inflammation [28] (Fig. 2). Similarly, dectin-1 signaling has been proven to be essential for the development of Japanese cedar pollen–induced allergic rhinitis in mice [30] and participates in lung inflammation in a murine model of fungal allergy through the induction of IL-22 [31]. By contrast, it has been demonstrated that invertebrate tropomyosin (for example, Der p 10 in HDM) binds to dectin-1 at the mucosal surfaces and mediates protection against allergic diseases [32]. In addition, it has been demonstrated that dectin-1 is downregulated in the epithelium of allergic patients due to the aberrant production of IL-33 [33]. In line with these results, Gu et al. have described that dectin-1 activation suppressed allergic type 2 responses [34]. Although many research efforts have been made in understanding the role of dectin-1 in the promotion or prevention of type 2 immune responses to allergens, further studies are still needed to fully elucidate its actual role in allergic diseases.

Dectin-2 is also involved in HDM-induced allergic airway inflammation as shown in different mouse models [35–37]. Notably, dectin-2-deficient mice display decreased eosinophilic and neutrophilic inflammation and Th2 responses in lung after HDM-induced sensitization [35]. Blocking of dectin-2 with specific antibodies inhibits HDM- as well as Der p 2-induced IL-5 and IL-13 production in cocultures of DC-T cells from asthmatic patients [38], which are characterized by an increased dectin-2 expression profile in peripheral blood mononuclear cells (Fig. 2) [39••].

Although DCIR is an inhibitory CLR, it has been involved in allergen processing and signaling. DCIR binds cockroach allergen Bla g 2 and mediates its uptake by human basophils and mast cells, leading to their activation and contributing to Th2 responses [40, 41]. In a mice model of atopic dermatitis, DCIR (*DCIR*^*−/−*^)–deficient mice exposed to Bla g 2 display an attenuation of allergen-induced skin inflammation compared to wild-type mice [41], suggesting that DCIR might well play a key role during skin sensitization and allergen-mediated inflammation in the context of atopic dermatitis.

MR has been involved in the recognition and internalization of different allergens from HDM (Der p 1 and Der p 2), dogs (Can f 1), cats (Fel d 1), cockroaches (Bla g 2), and peanuts (Ara h 1, Ara h 3) through their carbohydrate moieties [42–45]. MR-deficient DCs show a reduced capacity to capture these allergens, although other receptors could be also implicated (Fig. 2). Binding assays reveal that CTLD4-7 domains of MR mediate the binding of Der p 1, Der p 2, Can f 1, Ara h 1, and Bla g 2 [42]. However, Fel d 1 is a ligand of the MR cysteine-rich domain [45]. MR participates in the Th2 polarization imprinted by Der p 1 in DCs [42]. Similarly, a mice model of allergic rhinitis has showed that MR has an essential role in Th2 polarization induced by the airborne allergen Fel d 1 [45]. Interestingly, DCs from allergic patients express higher levels of MR and uptake Der p 1 more efficiently than DCs from healthy donors [43, 46]. On the other hand, MR-deficient lung macrophages have displayed reduced cockroach allergen uptake, and MR-deficient mice have showed increased lung inflammation and exacerbated Th2 responses, suggesting that MR might well also play a very important role in the attenuation of type 2 inflammatory responses to allergens in the airways [47]. Therefore, deeper investigations are still required to firmly elucidate the actual role of MR in the regulation of type 2 immune responses to allergens or tolerance induction within the context of different allergic diseases.

DC-SIGN has been also implicated in the immunopathology of allergy due to its capacity to recognize allergens from HDM (Der p 1), foods (Ara h 1), animal dander (Can f 1, Fel d 1), and pollens (BG-60) [48, 49]. The major peanut allergen Ara h 1 binds to DC-SIGN activating DCs and inducing the polarization towards Th2 cells [50]. It has been described that peanut agglutinin is also a ligand of DC-SIGN that activates DCs in vitro [51]. Other common allergenic foods such as soybean, tree nuts, chicken egg, and milk contain DC-SIGN-binding proteins, which have showed serum IgE-reactivity [51]. Allergens from HDM and chicken egg, Der p 2 and Gal d 2, are captured by human DCs through DC-SIGN binding and promote the priming of Th2 and Th22 responses [49]. The use of a DC-SIGN blocking antibody impairs Der p 2 and Gal d 2 uptake by DCs as well as Th2 and Th22 polarization [49]. It has also been reported that HDM allergen Der p 7 can activate DCs via DC-SING binding and lead to polarization of Th2 in vitro (Fig. 2) [52]. Native or heat-inactivated allergens from HDM extract are endocytosed through DC-SIGN, reducing DC-SIGN expression on the surface of DCs, but inducing signaling events that promote the generation of Th2 cells [53]. However, it has been also shown that DC-SIGN deletion in DCs promoted Th2 polarization in autologous cocultures of Der p 1–stimulated DCs with T cells, suggesting that the axis Der p 1-DC-SIGN might well also promote allergen-specific Th1 or regulatory responses able to impair type 2 polarization [54]. Under this scenario, it was previously described that Der p 1 due to its cysteine protease activity can cleave DC-SIGN but not MR, which might play also an important role in the amplification of type 2 immune responses, thus enhancing its allergenicity [55, 56]. Therefore, further studies are also needed to clarify the actual role of DC-SIGN in the context of allergic diseases within the different potential scenarios.

## Can We Exploit CLRs as Potential Therapeutic Targets for Allergic Diseases?

As above described, in addition to their role in the initiation of type 2 pro-inflammatory immune responses against encountered allergens, specific CLRs can also promote the generation of allergen-specific Th1 and/or Treg cells that might well contribute to the immunoregulation of allergic diseases and to the restoration of healthy immune responses to allergen. The final functional outcomes depend on the specific activated CLRs and on the integration of the initiated downstream signaling pathways, which is also finely tuned by the physical nature of the carbohydrate ligands as well as by the affinity and avidity of the carbohydrate-CLR engagement. Remarkably, it has been shown that depending on its 3D configuration, solubility, and stoichiometry, the same carbohydrate structure might well simultaneously activate different CLRs within the same cell [7, 27]. The final integration of all these signals will determine the final functional outcomes that can be achieved in the context of allergic diseases. At this regard, different studies conducted so far have shown that activation of specific CLRs, such as dectin-1, DCIR, DC-SIGN, or DEC205, induces anti-inflammatory responses that could alleviate allergic symptoms in patients [32, 57–60]. The potential immunoregulatory properties displayed by specific CLRs under certain conditions have been recently exploited to develop next-generation vaccines for AIT [12].

AIT is the only treatment for allergic diseases with the potential capacity to induce long-lasting disease modification. AIT is based on repeated administration of high doses of the causative allergen to achieve a state of long-term tolerance that persists after treatment discontinuation [61, 62]. Although many clinical trials and real-life experience demonstrated AIT as a safe and effective treatment for many patients, it still faces important drawbacks in terms of efficacy, safety, the long duration of the treatment, or the large number of administrations needed to induce tolerance, which is associated to low patient adherence [12, 61, 62]. In this regard, vaccines targeting DCs by coupling allergens to specific carbohydrate structures have been pointed as a suitable strategy that might well help to overcome these problems [10••, 12]. This approach targets allergen vaccines to specific immune cells (i.e., DCs) through specific CLRs, which might significantly increase the effective doses of the delivery allergens. Simultaneously, targeting specific CLRs with the conjugated carbohydrates elicits immunomodulatory responses in the same DCs, thus potentiating the capacity to induce allergen tolerance [10••, 12]. Until now, the beneficial effects of several carbohydrates conjugated with allergens have been reported for β-glucan, N-acetylgalactosamine, mannose, and mannan [13, 63–67, 68••, 69, 70].

The most clinically advanced strategies for glycan-allergen conjugates for AIT are the next-generation vaccines based on the use of polymerized allergens conjugated to non-oxidized mannan (allergoid-mannan conjugates).

**Fig. 3:**
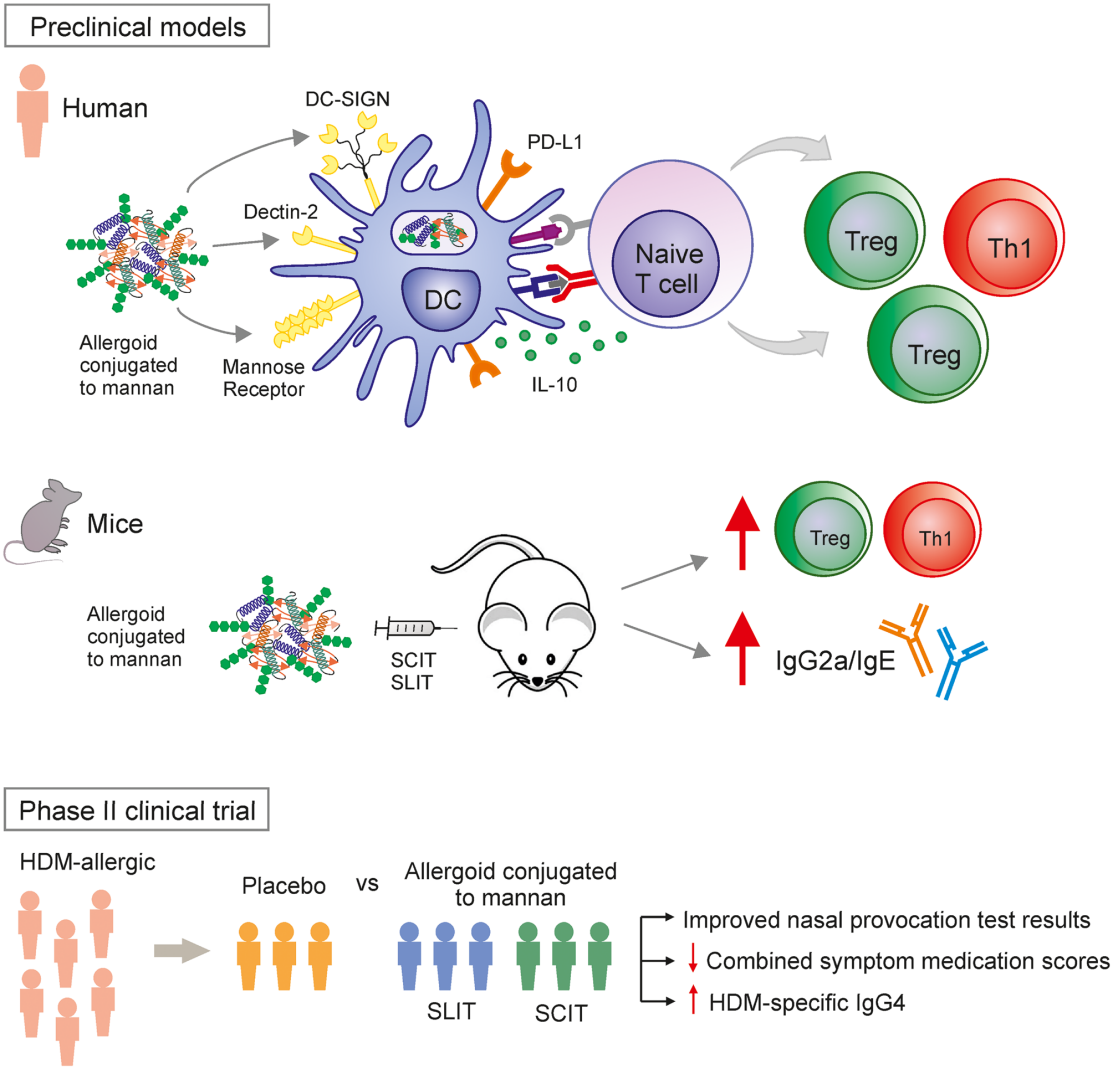
Polymerized allergens conjugated to non-oxidized mannan have been developed as allergen-specific immunotherapy (AIT) vaccines. Preclinical data in human and mice showed that polymerized allergens conjugated to mannan specifically target DCs inducing the acquisition of tolerogenic and anti-allergic features, thus enhancing Treg cell polarization and Th1 responses, as well as increasing IgE-blocking antibodies. A phase II clinical trial for the treatment of rhinitis/rhinoconjunctivitis demonstrated that a formulation against HDM allergy administered either subcutaneously or sublingually is safe and effective in achieving primary and secondary clinical outcomes. Primary outcome was the improvement of titrated nasal provocation test and secondary outcomes were the combination of symptom and medication scores and serological markers. DC, dendritic cells; PD-L1, programmed death ligand 1; DC-SIGN, DC-specific intercellular adhesion molecule 3-grabbing nonintegrin; Treg, regulatory T cell; SCIT, subcutaneous immunotherapy; SLIT, sublingual immunotherapy

As a proof of concept, initial preclinical studies demonstrated that allergoid-mannan conjugates from *P. pratense* grass pollen allergens target DCs via CLRs (MR, DC-SIGN, and dectin-2) significantly enhancing allergen uptake and promoting the generation of functional allergen-specific FOXP3^+^ Treg cells by mechanisms partially depending on IL-10 and PD-L1 (Fig. 3) [13, 71]. The tolerogenic properties imprinted by allergoid-mannan conjugates in DCs are driven by an enhanced production of ROS and a metabolic rewiring induced by mTOR signaling pathway consisting of an increased rate of glycolysis and lactate fermentation, which is impaired by the presence of aluminum, a commonly used adjuvant for AIT [72]. Similarly, allergoid-mannan conjugates impair the generation of pro-inflammatory and pro-allergic human macrophages, whereas they potentiate the polarization of macrophages with regulatory functions [69]. In mice, after subcutaneous or sublingual immunization, allergoid-mannan conjugates showed better results than native allergens or allergoids in terms of increasing IgG2a/IgE, IFN-γ/IL-4 ratios, IL-10-producing cells, and FOXP3^+^ Treg cells (Fig. 3) [13, 71]. Remarkably, differentiation of macrophages and DCs from human monocytes in the presence of allergoid-mannan conjugates also induces tolerogenic profiles, as indicated by the high expression of the tolerogenic molecules IL-10, indoleamine 2,3-dioxygenase (IDO), PD-L1, suppressor of cytokine signaling (SOCS) 1, and SOCS3. In these freshly generated cells, lipopolysaccharide stimulation triggers a reduced release of the pro-inflammatory cytokines IL-6 and TNF-α, and promotes an increase of the IL-10/TNF-α ratio and a greater capacity to induce functional FOXP3^+^ Treg cells [68••, 69]. The acquisition of this tolerogenic phenotype is supported by a metabolic and epigenetic reprogramming [68••]. In this scenario, glucose metabolism is switched from Warburg effect and lactate production to mitochondrial oxidative phosphorylation. In addition, an epigenetic reprogramming within tolerogenic loci, lower expression levels of histone deacetylase genes, increased expression of the anti-inflammatory miRNA-146a/b, and decreased pro-inflammatory miRNA-155 contribute to the induction of this phenotype [68••].

Interestingly, in a phase II clinical trial enrolling 196 HDM-allergic patients, it has been recently published that allergoid-mannan conjugates, either subcutaneously or sublingually administered, display efficacy and safety for the treatment of allergic rhinitis/rhinoconjunctivitis (Fig. 3) [14••]. There are also other ongoing phase II clinical trials to assess the efficacy and safety of allergoid-mannan conjugates from grass (NCT02654223) and birch (EudraCT: 2018–002,522-23 & 2020–004,126-32) pollen for the treatment of allergic rhinitis/rhinoconjunctivitis, which results are expected to be published soon. In the same way, phase III clinical trials for allergoid-mannan conjugates are currently ongoing for the treatment of patients with mild to moderate HDM-induced asthma and rhinitis/rhinoconjunctivitis (NCT05400811) or birch pollen–induced allergic rhinitis/rhinoconjunctivitis (EudraCT: 2021–002,252-36). Results from these ongoing clinical trials might well help to definitely determine if these next-generation vaccines targeting CLRs display clinical benefits that can be translated into real-life improvements of allergic patients’ quality of life.

## Conclusion and Future Perspectives

Cellular and molecular mechanisms involved in the generation of type 2 immune responses to allergens have been deeply studied over the last years. Recent findings uncovered that allergens contain carbohydrate structures that are recognize by specific CLRs, which in turn activate different signaling pathways on the targeted cells, thus leading to the generation of allergen-specific CD4^+^Th2 cells. Under different conditions, specific CLRs might well promote tolerogenic responses to allergens. A better knowledge on the actual carbohydrate fraction of allergens that interact with specific CLRs and how signaling pathways are orchestrated depending on the stoichiometry, affinity, and avidity of this binding is still needed. Carbohydrate-modified allergens might represent the next-generation vaccines targeting specific immune cells for AIT. At this regard, allergoid-mannan conjugates targeting DCs enhance the allergen uptake and promote healthy immune response to allergens. Allergoid-mannan conjugated vaccines for grass pollen, birch pollen, and HDM allergy have been already developed and assayed in phase II clinical trials and phase III clinical trials are currently ongoing. Overall, CLRs represent suitable targets for AIT but the better understanding of the mechanisms underlying the mode of action of superfamily of receptors at the molecular level in the context of allergic diseases might well open new opportunities for the development of future therapeutic approaches in the field of allergy.

## Abbreviations

AIT: Allergen-specific immunotherapy
CLRs: C-type lectin receptors
CTLD: C-type lectin-like domain
DAP12: DNAX-activation protein 12
DCIR: DC immunoreceptor
DCs: Dendritic cells
DC-SIGN: DC-specific intercellular adhesion molecule 3-grabbing nonintegrin
FcR: Fc receptor
FcRγ: Fc receptor γ
FcεRI: High-affinity IgE receptor
HDM: House dust mite
HmoDCs: Human monocyte-derived DCs
IDO: Indoleamine 2,3-dioxygenase
Ig: Immunoglobulin
ILC2: Type 2 innate lymphoid cells
ITAM: Immunoreceptor tyrosine–based activating motif
ITIM: Immunoreceptor tyrosine–based inhibitory motif
MR: Mannose receptor
NF-κB: Nuclear factor-κB
PD-L1: Programmed death ligand 1
PM: Polymerized allergens conjugated to non-oxidized mannan
PRRs: Pattern recognition receptors
ROS: Reactive oxygen species
SCIT: Subcutaneous immunotherapy,
SHP: SH2‑domain‑containing protein tyrosine phosphatase
SLIT: Sublingual immunotherapy
SOCS: Suppressor of cytokine signaling
Syk: Spleen tyrosine kinase
TLRs: Toll-like receptors
Treg: Regulatory T
